# Artificially Engineered Synthetic Biomarkers Revolutionizing Early Diagnosis of Diseases

**DOI:** 10.3390/molecules30234532

**Published:** 2025-11-24

**Authors:** Anyun Wang, Min Wei, Keying Yu, Siying Heng, Xinyue Zhao, Wenxi Jian, Jinsong Zhao, Yanfeng Gao, Yanping Wang

**Affiliations:** 1 School of Public Health, Wannan Medical College, Wuhu 241002, China; 20060016@wnmc.edu.cn; 2 School of Basic Medical Sciences, Wannan Medical College, Wuhu 241002, China; 20240013@wnmc.edu.cn; 3 School of Medical Imaging, Wannan Medical College, Wuhu 241002, China; yu_ke_ying@163.com (K.Y.); hengsiying@163.com (S.H.); zhaoxinyue_2005@163.com (X.Z.); jianwenxi_wnmc@163.com (W.J.)

**Keywords:** synthetic biology, exogenous biomarkers, artificially engineered biomarkers, cancer, disease diagnosis, point-of-care testing, precision medicine

## Abstract

Early detection of disease, particularly cancer, is pivotal for improving patient outcomes. However, diagnostic approaches relying on traditional endogenous biomarkers often face significant limitations, primarily due to the low abundance and lack of specificity of these markers, which can lead to delayed or inaccurate diagnosis. Synthetic biomarkers, artificially engineered exogenous agents that are designed to interact with specific biological targets to generate detectable signals, represent a revolutionary approach in diagnostic medicine. By allowing for the conversion and amplification of specific disease-related signals, synthetic biomarkers enable accurate diagnosis, prognosis, and therapy monitoring, holding immense promise for the early detection of diverse disease states to facilitate prompt treatment initiation and to improve clinical outcomes. This review provides a comprehensive overview of the evolution, classification, working mechanisms, and diagnostic applications of synthetic biomarkers, along with a discussion of their future potential in advancing precision medicine and effective disease management.

## 1. Introduction

Early detection of diseases, particularly cancer, is crucial for effective diagnosis, treatment, and prognosis. Identifying a disease in its nascent stages, before the onset of clinical symptoms, enables the adoption of less invasive and more effective treatment strategies. This approach minimizes side effects, increases survival rates, improves long-term quality of life, and reduces overall healthcare costs [1,2,3]. For example, the mortality rate for breast cancer can be reduced by up to 50% when the disease is detected at an early stage compared with advanced stages [4,5]. Various technologies have been developed for early disease detection based on traditional endogenous biomarkers, such as cells, extracellular vesicles, proteins, nucleic acids, and metabolites [6,7,8,9]. However, achieving highly efficient and accurate early diagnosis based on endogenous biomarkers remains challenging, predominantly due to the lack of highly specific biomarkers, the low sensitivity of available markers, and their variable expression influenced by general factors, such as age, gender, and lifestyle [10,11,12].

The concept of synthetic biomarkers has emerged as a transformative strategy in disease diagnostics, heralding a new era of precision medicine [13]. These biomarkers are artificially engineered at the molecular or cellular level and are administered as exogenous agents. They are designed to interact with specific biological targets [14] or disease-related conditions [15], to reveal critical information about physiological or pathological states. Being capable of generating amplified signals, particularly in scenarios where endogenous biomarkers are insufficient, synthetic biomarkers not only overcome the limitations of traditional markers but also enable highly precise and individualized healthcare diagnostics with unparalleled specificity and sensitivity [16,17,18,19].

**Figure 1:**
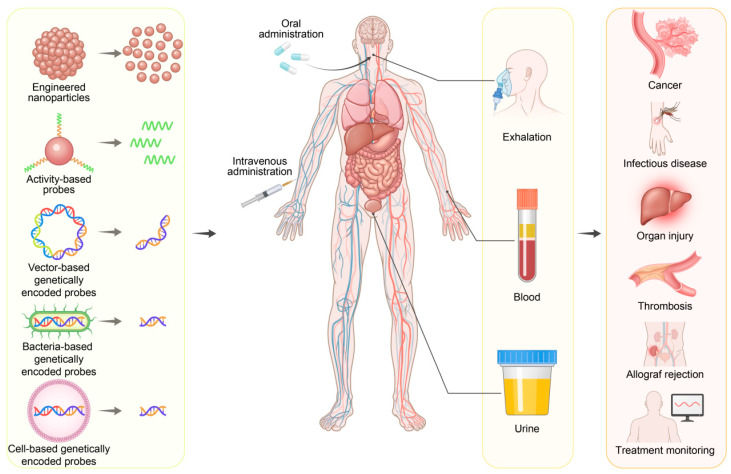
Schematic illustration of synthetic biomarkers in the early detection of a variety of diseases.

In recent years, there have been significant breakthroughs and rapid developments in novel and highly effective technologies for early disease detection based on synthetic biomarkers. In this review, we aim to illuminate the significant advancements in synthetic biomarkers for the early diagnosis of cancers and other diseases. We begin with a comprehensive exploration of the discovery and evolution of synthetic biomarkers. Next, we will present a variety of synthetic biomarkers categorized by their mechanisms of action. Subsequently, we will examine their applications in disease diagnosis, highlighting their superiority over traditional endogenous biomarkers. The review concludes with a discussion of cutting-edge developments, future directions, and the challenges that lie ahead in this burgeoning field (Figure 1).

## 2. Advent of Synthetic Biomarkers

**Figure 2:**
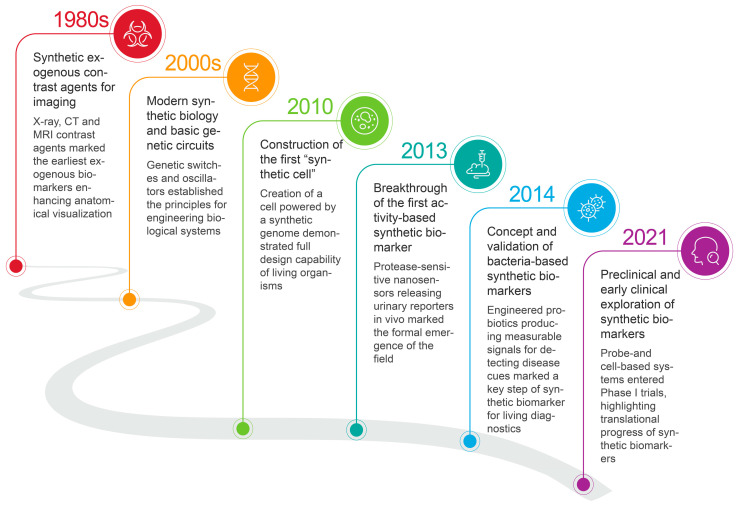
Historical development of synthetic biomarkers.

The history of exogenous synthetic biomarkers is a compelling story of interdisciplinary innovation, driven by advances in chemistry, biology, genome editing, and engineering (Figure 2). The initial use of synthetic compounds in diagnostics dates back to the 1980s, when they were applied as contrast agents in medical imaging, such as magnetic resonance imaging (MRI), computed tomography (CT), and positron emission tomography (PET) [20,21,22,23]. These foundational agents enhance the visualization of specific tissues and abnormalities, setting the stage for more sophisticated approaches to disease detection.

The true paradigm shift, however, was enabled by the rapid advancement of nanotechnology and genome editing in diagnostic applications at the beginning of the 21st century [24,25,26]. Over the past few decades, the ability to engineer nanomaterials with unique properties has broadened the horizons for creating molecules capable of mimicking or interacting with biological systems [27,28]. This innovation paved the way for the concept of modern “synthetic biomarkers,” pushing it into a distinct field. A pivotal moment came in 2013 with the groundbreaking work of Sangeeta N. Bhatia’s team at the Massachusetts Institute of Technology, which pioneered the use of engineered molecular agents for early detection of cancer [29]. Their research introduced a revolutionary class of protease-sensitive nanoprobes that, upon cleavage in diseased tissues, release mass-encoded peptides into the urine for noninvasive detection via mass spectrometry. This work illuminates the potential of these novel probes to overcome the limitations of traditional diagnostics and highlights the official birth of this burgeoning field.

Since this breakthrough, the development of synthetic biomarkers has been propelled by the urgent need for early disease diagnosis and the rise of precision medicine [30,31,32,33]. A wide variety of synthetic biomarkers have been developed, overcoming the limitations of their natural counterparts by amplifying minute signals, enabling noninvasive and highly sensitive detection, which has demonstrated superior performance and broad application prospects in the early diagnosis of a variety of major diseases.

## 3. Mechanistic Categories of Synthetic Biomarkers

By leveraging advanced engineering and molecular design, synthetic biomarkers can be broadly categorized into several fascinating classes, including engineered nanoparticles, activity-based probes, and genetically encoded reporters. Each of these categories employs unique mechanisms to generate clear, quantifiable signals of disease presence, progression, or response to treatment. This diversity in design highlights the transformative potential of synthetic biomarkers in revolutionizing disease diagnostics.

### 3.1. Engineered Nanoparticles

Nanoparticles (NPs) are ultrasmall particles with sizes typically between 1 and 100 nm that possess unique physical, chemical, and biological properties, such as large surface areas, tunable surface chemistry, and size-dependent light absorption/emission [34,35,36,37]. These attributes, combined with their good biocompatibility, degradability, facile functionalization, ability to cross biological barriers and efficient renal clearance potential, make NPs excellent candidates for biomedical applications, such as drug delivery and controlled release, biosensing and diagnostics, and targeted disease therapy [38,39,40,41].

**Figure 3:**
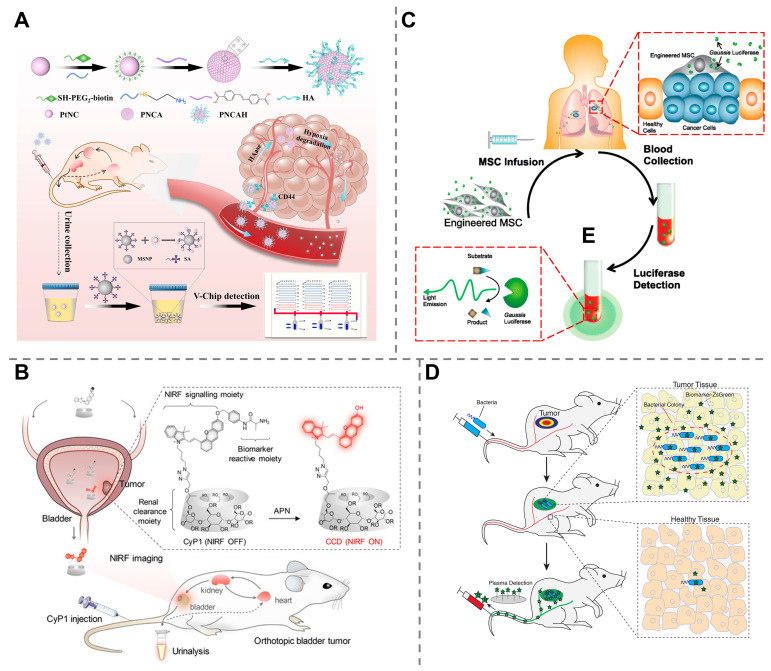
Various synthetic biomarkers with different working principles. (**A**) Hypoxia-responsive platinum supernanoparticles enabling noninvasive urinary monitoring of triple-negative breast cancer and lung metastasis via a V-Chip. Reprinted with permission from Ref. [49]. Copyright 2022, Wiley-VCH GmbH. (**B**) APN-activated ABP by linking an APN-cleavable peptide, a fluorophore and a renal clearance moiety (2-hydroxypropyl)-b-cyclodextrin for bladder cancer detection. Reprinted with permission from Ref. [50]. Copyright 2020, Wiley-VCH GmbH. (**C**) MSCs engineered to express and secrete hGluc as a reporter of cancer for cancer diagnosis and monitoring. Reprinted with permission from Ref. [51]. Copyright 2015, BioMed Central Ltd. (**D**) *Salmonella* bacteria genetically modified to express and release ZsGreen for evaluation of the presence and size of tumor through blood biopsy. Reprinted with permission from Ref. [52]. Copyright 2020, Wiley-VCH GmbH.

Through deliberate design, NPs can be engineered to be functionalized with targeting ligands, such as antibodies or peptides, to bind selectively to specific biomolecules at diseased sites or within the tumor microenvironment, which is crucial for early and accurate detection [42,43,44,45]. Upon binding, the NPs can further interact with disease-associated pathological conditions to generate a detectable signal, probably from their intrinsic properties (e.g., colorimetric changes in gold NPs and peroxidase-like enzymatic activity of platinum NPs) [46]. The generated signal is then detected via various imaging modalities or analytical techniques, such as fluorescence imaging [47] or body fluid tests [32]. For example, by conjugating AuNPs with a caspase-1 enzyme-cleavable fluorescent peptide (FP_1_Au) and modifying them with apoptotic bodies (ABs), a smart engineered nanoprobe (FP_1_Au@AB) was developed. The ABs helped macrophages recognize and eat these nanoprobes, after which the peptides were cleaved by caspase-1, an important biomarker of intracellular bacterial infection, resulting in fluorescence signals for diagnosis. This synthetic biomarker effectively detected suppurative arthritis via the synovial fluid of mice [48]. Our group has also reported the development of hypoxia-responsive platinum supernanoparticles that dissociate into ultrasmall platinum nanoclusters (PtNCs) in the tumor microenvironment, enabling noninvasive urinary monitoring of triple-negative breast cancer and lung metastasis via a volumetric bar-chart chip (V-Chip). This approach detected tumors as small as ~50 mm^3^ in size in mice with high sensitivity (AUC = 0.93–0.95) and successfully monitored treatment response in real time (Figure 3A) [49].

Overall, through precise engineering, NPs serve as highly versatile and functional platforms for advanced biomedical detection and diagnostics, leveraging their tunable properties and targeted design to enable sensitive and specific identification of disease biomarkers.

### 3.2. Activity-Based Probes

Biological activities, such as specific enzyme functions, signaling pathway dysregulation and metabolic changes, are strongly associated with abnormal physiological conditions in vivo [53,54,55,56]. An activity-based probe (ABP) [57] forms another group of important synthetic biomarkers specially designed to transform the disease-related biological activity into a detectable signal, rather than simply detecting the presence of a biomolecular marker [57,58].

The most investigated ABP is a protease-activated probe. Proteases, or enzymes that break down proteins and peptides by cleaving the peptide bonds between amino acids, are crucial for a variety of physiological processes, including digestion, immune response, cell signaling, and apoptosis. Their dysregulated activity is a key indicator of diseases, such as cancer, infectious diseases and neurodegenerative disorders [59,60,61]. Protease-activated probes are typically designed as a modular system consisting of a substrate, a reporter molecule (e.g., a fluorophore), and a quencher. In its inactive state, the quencher is positioned close to the reporter, suppressing its signal. Upon interaction with the target protease, the substrate is specifically recognized and cleaved by a target protease, spatially separating the reporter from the quencher [62,63]. This cleavage event “activates” the probe, leading to the generation of a strong, detectable signal, such as fluorescence signals, which can be quantified to monitor real-time protease activity. For example, an ABP responsive to aminopeptidase N (APN), a critical indicator of tumor invasion, angiogenesis, and metastasis, was developed by linking an APN-cleavable peptide, a fluorophore and a renal clearance moiety (2-hydroxypropyl)-b-cyclodextrin. Upon reaction with APN, the peptide was cleaved and the fluorophore was activated, allowing for near-infrared fluorescence imaging and urinary fluorescence assays to detect bladder cancer in mice (Figure 3B) [50].

By revealing abnormal patterns of activity expression, ABPs provide dynamic, functional insights into disease progression, which allows for the effective identification of diseases and organ dysfunction at very early stages. This ability to provide real-time functional information makes ABPs highly promising for the development of novel diagnostic tools that go beyond traditional static biomarkers.

### 3.3. Genetically Encoded Probes

Genetically encoded probes represent a novel class of engineered biomolecule design that has emerged with the advancement of gene editing techniques [64,65]. The core principle involves genetically modifying living carriers, such as viruses [66], mammalian cells [67] and bacteria [68,69], to carry a reporter gene that produces a detectable signal only when activated by disease-specific stimuli. Upon administration, the carriers seek out and accumulate at the disease site, where local molecular triggers activate the expression of the reporter, generating measurable signals, such as fluorescence or luminescence, for external detection. These synthetic biomarkers can be broadly classified based on the type of carrier used, including vector-based, mammalian cell-based, and bacteria-based biomarkers.

Vector-based synthetic biomarkers operate by using viral or nonviral vectors to carry disease-selective promoters that drive the expression of a synthetic biomarker. These biomarkers can be produced based on the producer cell (e.g., human embryonic kidney 293 cells) followed by purification and verification. An adenovirus serotype 5 vector was engineered to incorporate a tumor-selective MUC1 promoter and a firefly luciferase reporter gene, acting as a targeted delivery vehicle for transporting genetic sequences into breast cancer cells. Upon entering breast cancer cells, the MUC1 promoter was activated, initiating the expression of the reporter gene and resulting in the emission of a detectable luminescent signal that can be monitored externally for tumor detection [70]. Viral vectors provide efficient gene delivery and long-term expression but raise safety concerns related to insertional mutagenesis, pre-existing immunity, and dosage control.

Mammalian cell-based synthetic biomarkers have been developed by using mammalian cells as diagnostic vehicles, as they can easily target and infiltrate tumor tissue. For example, mesenchymal stem cells (MSCs) could be engineered to express and secrete humanized Gaussia luciferase (hGluc) as a reporter of cancer. When systemically administered into the body and selectively homed to tumor sites, hGluc was continuously released into the bloodstream, which was further measured for cancer diagnosis and monitoring (Figure 3C) [51]. Mammalian cell-based systems enable precise sensing and response within the physiological environment. However, their widespread use is limited by scalability, stability, and manufacturing complexity.

Bacteria-based synthetic biomarkers work based on genetically modified bacteria that can selectively colonize tumor sites and express and secrete detectable reporters for diagnostics. Typically, these bacteria-based biomarkers can be manufactured by using clinically validated chassis strains with integrated safety circuits and standardized encapsulation or lyophilization for formulation. For example, *Salmonella* bacteria were genetically modified to express and release a fluorescent protein called ZsGreen as a synthetic biomarker. These bacteria could continuously produce ZsGreen at the tumor site, allowing the detection of tumors weighing only 120 mg in a mouse model through blood biopsy (Figure 3D) [52]. Bacterial carriers offer advantages, such as easy genetic manipulation, low production cost, and potential for oral or localized delivery, but face challenges in biosafety control, immunogenicity, and regulatory classification.

Collectively, these genetically encoded systems represent a powerful platform for next-generation diagnostics. By turning the body’s own cells or introducing microorganisms into sophisticated biosensors, they offer an unprecedented level of specificity and signal amplification, paving the way for highly sensitive and minimally invasive disease monitoring.

Overall, different categories of synthetic biomarkers exhibit distinct strengths and limitations (Table 1). Engineered nanoparticles feature scalable synthesis and flexible signal amplification but face challenges related to batch reproducibility, long-term safety, and regulatory approval. Activity-based probes offer exceptional sensitivity and noninvasive detection but are constrained by enzyme specificity and substrate design. Genetically encoded probes provide programmable sensing and real-time monitoring yet raise biosafety and immunogenicity concerns, requiring rigorous preclinical evaluation.

Taken together, each category holds promise for advancing precision diagnostics, and the precise selection of careful design of synthetic biomarkers based on the biological targets and the diagnostic mechanism is essential. Moreover, their translation to clinical use will depend on overcoming technical, regulatory, and manufacturing barriers, underscoring the need for continued multidisciplinary innovation.

## 4. Applications of Synthetic Biomarkers in Disease Diagnosis

The unique characteristics of synthetic biomarkers, such as high specificity and signal amplification capability, combined with the use of minimally invasive detection modalities, enable their wide application in disease diagnosis to provide earlier, more accurate, and less invasive diagnostic insights. In this section, we will explore the pivotal applications of synthetic biomarkers in the early detection and management of major diseases, following a brief description of the adaptive detection methods.

### 4.1. Detection Modalities of Synthetic Biomarkers

Depending on the molecular design, chemical stability, and route of administration, synthetic biomarkers can be excreted or detected in body fluids, such as urine, blood, and saliva, through breath, or by imaging techniques. While slightly invasive, blood-based detection remains the gold standard for quantitative biomarker analysis [71,72]. Engineered probes, such as nanoparticles and activity-based probes, can be designed to release small, cleaved reporters or peptides into the bloodstream upon encountering a disease target, which could be further measured via traditional immunoassays or novel microfluidic platforms [73,74]. Urine represents another widely exploited matrix for liquid biopsy due to its easy accessibility, noninvasiveness, and minimal interference from the complex plasma environment [75,76]. Many ABPs and engineered NPs are designed to release reporter fragments that are small enough to be cleared by the kidneys, offering high temporal resolution and facilitating continuous monitoring of disease progression. In addition, breath analysis, as a noninvasive method, works based on the detection of volatile organic compounds (VOCs) released in relation to specific diseases [77]. APBs, for instance, can be engineered to be cleaved by lung-specific enzymes and alter the exhaled VOC profiles, which can be further detected using sensitive analytical techniques like electronic noses [78], offering potential for diagnosing lung cancer, gastrointestinal disorders, and metabolic diseases. Furthermore, imaging techniques, providing spatial and anatomical context, are also widely employed to turn the detection into localization [79]. Engineered NPs tagged with imaging agents (e.g., fluorophores) can be designed to accumulate at the disease site in order to allow for the direct visualization of tumors, inflammatory lesions, or infected tissues using modalities like fluorescence imaging [80,81,82]. The key advantage is the ability to locate the disease precisely, monitor its size, and guide interventions, such as surgery.

### 4.2. Early Detection of Cancers

Early detection of cancer significantly improves treatment outcomes and survival rates. However, traditional methods relying on endogenous biomarkers, such as circulating tumor cells (CTCs) or cell-free tumor DNA (cfDNA), frequently encounter significant limitations stemming from their low abundance and rapid clearance from the bloodstream. Synthetic biomarkers provide a revolutionary solution by employing engineered probes that generate amplified, easily detectable signals, thus facilitating the accurate diagnosis of malignancies at their earliest stages.

**Figure 4:**
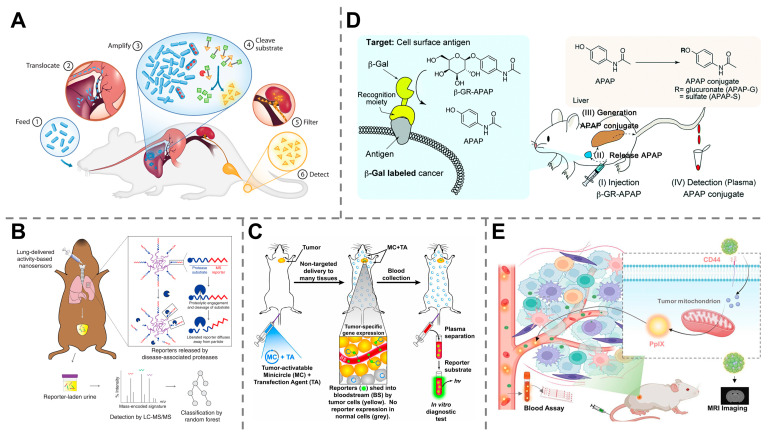
Synthetic biomarker-based technologies for early diagnosis of cancers. (**A**) *E. coli* Nissle 1917 bacteria-based synthetic biomarker for urinary detection of liver metastases in mice. Reprinted with permission from Ref. [84]. Copyright 2015, American Association for the Advancement of Science. (**B**) Protease activity-based nanosensors for detecting lung adenocarcinoma in mice. Reprinted with permission from Ref. [85]. Copyright 2020, The Authors. (**C**) Nonviral vector-based synthetic biomarkers carrying fluorescence reporter genes for metastatic melanoma diagnosis in mice through blood analysis. Reprinted with permission from Ref. [92]. Copyright 2015, National Academy of Sciences. (**D**) β-gal-responsive prodrug for the targeted detection of tumors via plasma analysis. Reprinted with permission from Ref. [93]. Copyright 2018, Royal Society of Chemistry. (**E**) A cancer-targeted nanoassembly that released synthetic biomarkers for cancer monitoring by using a microfluidic chip. Reprinted with permission from Ref. [94]. Copyright 2025, American Chemical Society.

Using urine for a “liquid biopsy” offers a noninvasive diagnostic approach, allowing for painless and real-time monitoring of cancer progression. However, its utility is generally limited to urological cancers, such as bladder, prostate and renal cancers, whereas biomarkers from other cancerous sites are not easily shed into the urine. Synthetic biomarkers, which are engineered to target tumors elsewhere in the body and release detectable reporters that are excreted into the urine, overcome this constraint and make this convenient sampling method applicable to a wide range of nonurinary cancers [83]. In a seminal work conducted by Sangeeta N. Bhatia, the probiotic bacterium *E. coli* Nissle 1917 was programmed and engineered to express the enzyme β-galactosidase. When administered orally, these bacteria selectively colonized liver metastases in mice and produced a detectable signal in urine after systemic injection of a luciferin–galactose substrate, allowing the detection of tumors as small as 1 mm in diameter (Figure 4A) [84]. Later, the same group developed a multiplexed library of 14 protease activity-based nanosensors that, when delivered via intratracheal instillation, detect lung adenocarcinoma in mouse models through urinary readout. This approach achieved 100% specificity and up to 95% sensitivity, detecting tumors with volumes as small as ~2.78 mm^3^ in a mouse model, and successfully distinguished malignant lesions from benign lung inflammation (Figure 4B) [85]. Although synthetic biomarkers do not directly reflect the levels of traditional endogenous biomarkers, their ability to identify tumors smaller than those detectable by conventional methods indicates the potential for achieving superior detection limits. The success of these synthetic biomarkers of different categories in detecting nonurinary cancers through urinary readouts highlights the remarkable versatility of this technology, which is also tailored for the early diagnosis and treatment monitoring of numerous other malignancies [86,87,88,89,90,91].

While urinary detection offers a noninvasive and convenient diagnostic method, synthetic biomarkers are also effectively utilized in other biological fluids, such as blood, to enable early cancer diagnosis. For example, an innovative type of nonviral tumor-activatable minicircle carrying a fluorescence reporter gene (human secreted embryonic alkaline phosphatase) has been developed to generate detectable fluorescence signals in blood after systemic administration into tumor-bearing mice. This approach enabled the differentiation of mice suffering from metastatic melanoma from healthy subjects with high specificity at early stages of growth (Figure 4C) [92]. A beta-galactosidase (β-gal)-responsive small biomolecule prodrug was developed to release detectable acetaminophen upon reaction with β-gal at tumor sites. The released acetaminophen was then metabolized into conjugates that can be detected in plasma, resulting in ultra-highly sensitive identification of tumors (Figure 4D) [93]. Our group also developed a cancer-targeted nanoassembly responsive to CD44 and tumor-associated enzymes within the TME that released protoporphyrin IX into the blood as a synthetic biomarker. By detecting the synthetic biomarkers within a microfluidic chip, this method achieved quantitative monitoring of cancer in mouse models (Figure 4E) [94].

Taken together, these studies demonstrate the immense potential of synthetic biomarkers to achieve early cancer diagnosis across a diverse range of tumor types. By converting microscopic, localized disease signals into macroscopically detectable readouts, from protease-activated probes in urine to nanosensors in blood, this technology overcomes the inherent limitations of traditional endogenous biomarkers and heralds a new era in diagnostics that promises to enable the earlier, more precise discovery and treatment of cancer.

### 4.3. Detection of Infectious Diseases

Infectious diseases, typically caused by pathogens, such as viruses, bacteria and fungi, pose a major global public health challenge, and their rapid, accurate diagnosis is crucial for effective treatment and containment [95,96,97]. Traditional diagnostic methods, such as microbial cultures and polymerase chain reaction (PCR), are often time-consuming and rely on specialized laboratory facilities, which poses a significant obstacle in resource-limited settings or during outbreaks requiring a rapid response [98,99]. Furthermore, many existing biomarkers may lack the specificity or sensitivity needed for reliable detection at the earliest stages of disease. Synthetic biomarkers, which are capable of translating disease information into an amplified detectable signal, have therefore provided a powerful new approach to overcome these limitations.

**Figure 5:**
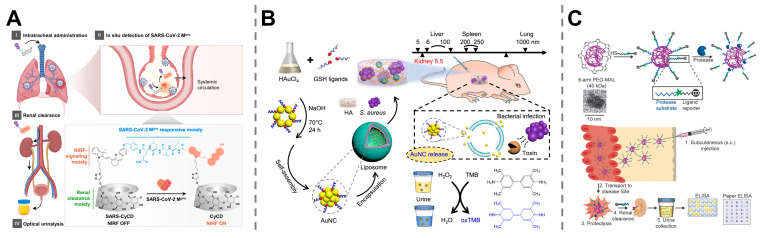
Application of synthetic biomarkers in the detection of infectious diseases. (**A**) Protease-activatable probes for noninvasive detection of SARS-CoV-2. Reprinted with permission from Ref. [100]. Copyright 2021, American Chemical Society. (**B**) Engineered gold nanoclusters for the effective detection of *Staphylococcus aureus* infection. Reprinted with permission from Ref. [101]. Copyright 2024, Springer Nature. (**C**) Protease activity-based probe for the quantification of *P. aeruginosa* lung infection. Reprinted with permission from Ref. [102]. Copyright 2016, Wiley-VCH GmbH.

Synthetic biomarkers have proven to be effective tools for rapid assessment of viral activities in the body, which has been crucial for the early diagnosis and control of viral infections during the COVID-19 pandemic. For example, Pu et al. reported a protease-activatable probe for noninvasive detection of SARS-CoV-2. The probe consisted of a hemicyanine fluorophore, a cyclodextrin unit, and a peptide substrate specific to the target protease that would be cleaved in response to the main protease of SARS-CoV-2, releasing a small, fluorescent fragment to generate detectable signals through optical urinalysis, allowing for highly sensitive and real-time monitoring of viral replication activity (Figure 5A) [100].

The utility of synthetic biomarkers extends beyond viral threats, offering a powerful platform for the rapid and specific detection of bacterial pathogens. A synthetic biosensor composed of engineered nanoparticles has been developed by encapsulating gold nanoclusters (AuNCs) within liposomes for effective detection of bacterial infection. Upon exposure to toxins secreted by pathogenic bacteria, the nanoparticles were ruptured to release AuNCs into the urine, where they catalyze a colorimetric reaction and produce a visible blue change for indicating *Staphylococcus aureus* infection (Figure 5B) [101]. In other studies, protease-sensitive synthetic biomarkers were developed for noninvasive diagnosis of lung infection. For example, poly(ethylene glycol) (PEG) scaffolds were used to link peptide sensitive to disease-associated proteases and fluorescein, a biotin reporter, to form administrable probes, which would be cleaved in the presence of the target protease and release the reporters. These reporters are cleared by kindness into the urine, followed by a quantification to reveal the degree of lung inflammation caused by *Pseudomonas aeruginosa* infection (Figure 5C) [102]. Similarly, another activity-based nanosensor was designed to be made of a polyethylene glycol core conjugated with a specific peptide substrate that could be cleaved by pathogen- and host-secreted proteases. Once injected, these nanosensors release detectable reporters into the urine in response to protease activity, allowing for the detection of *Pseudomonas aeruginosa* lung infections, monitoring infection progression, and evaluating antibiotic therapy efficiency [103].

Together, these works showcase how synthetic biomarkers are transforming infectious disease diagnostics through enhanced specificity, programmability, and deployability. These innovations highlight the versatility of synthetic biomarkers in enhancing infectious disease diagnostics, particularly in resource-limited settings where rapid, accurate, and accessible testing is essential.

### 4.4. Monitoring of Organ Dysfunction and Injury

Early detection of organ injury is crucial for effective treatment and improved patient outcomes. In conditions like acute kidney injury (AKI), for example, timely diagnosis can prevent progression to chronic kidney disease or even organ failure [104,105]. However, existing diagnostic methods based on traditional biomarkers, such as serum creatinine, frequently work after the occurrence of organ damage and often suffer from low specificity [106,107]. The emergence of synthetic biomarkers provides a powerful new tool to overcome these challenges. By designing engineered probes that respond to specific disease signals and translate them into easily detectable reporters, we can achieve noninvasive monitoring of organ dysfunction and injury at very early stages, often before clinical symptoms appear.

**Figure 6:**
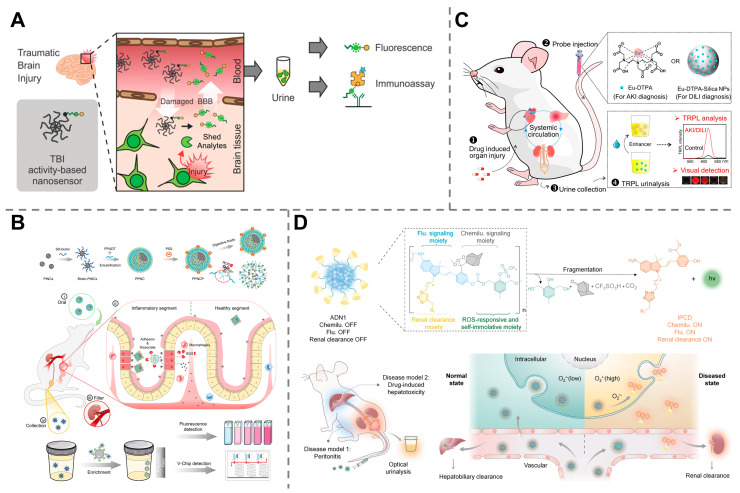
Application of synthetic biomarkers in the monitoring of organ injuries. (**A**) An activity-based nanosensor for noninvasive detection of traumatic brain injury. Reprinted with permission from Ref. [108]. Copyright 2023, Wiley-VCH GmbH. (**B**) Orally administered platinum nanomarker superparticles enabling ROS-responsive monitoring of IBD. Reprinted with permission from Ref. [109]. Copyright 2022, American Chemical Society. (**C**) Engineered Eu-DPTA-integrated silica NPs for the diagnosis and monitoring of drug-induced liver injury and acute kidney injury. Reprinted with permission from Ref. [110]. Copyright 2023, American Chemical Society. (**D**) Polymer-based nanoreporters for chemiluminescence diagnosis of drug-induced hepatotoxicity. Reprinted with permission from Ref. [111]. Copyright 2023, Wiley-VCH GmbH.

The promise of synthetic biomarkers for organ health monitoring is realized through several key platforms; for example, a great variety of findings utilize urine as a simple readout for evaluating organ functions. An activity-based nanosensor equipped with a calpain substrate peptide, a fluorescein fluorophore, and a CPQ2 quencher was capable of noninvasive detection of traumatic brain injury (TBI). Upon reaching injured brain tissue, the nanosensor was cleaved by the TBI-associated protease calpain, and the fluorophore was released into urine, generating a detectable fluorescence signal. Compared with the existing biomarker glial fibrillary acidic protein, this technology demonstrated enhanced sensitivity for detecting mild TBI (Figure 6A) [108]. Our group has reported that orally administered, ROS-responsive platinum superparticles (PPNCPs) could release catalytic platinum nanoclusters (PtNCs) in the inflamed gut, enabling noninvasive urinary monitoring of inflammatory bowel disease (IBD) with a detection limit in the micromolar range for H_2_O_2_ (responsive from 50 to 100 μM), significantly outperforming the clinical standard fecal calprotectin (fCAL) ELISA test (Figure 6B) [109]. Another similar work based on engineered nanoparticles has been reported based on the europium complexes of diethylenetriaminepentaacetic acid (Eu-DTPA) and Eu-DPTA-integrated silica nanoparticles, which were injected into mice, activated in the presence of organ injury, filtered by the kidneys into the urine, and detected via time-resolved photoluminescence. This approach enabled the diagnosis and monitoring of drug-induced liver injury and acute kidney injury at a very early stage when traditional blood assays remained inaccessible (Figure 6C) [110]. In some other works, polymer-based nanoreporters were designed to be activated by superoxide anions at a disease site, which induced smaller and renally clearable agents into the urine for chemiluminescence diagnosis of drug-induced hepatotoxicity (Figure 6D) [111] or near-infrared fluorescence imaging of hepatic ischemia–reperfusion injury [112].

Similarly, breath analysis has emerged as a promising diagnostic method for tracking pathologies in the lungs [113,114]. For example, a synthetic breath biomarker has been created based on the protease-activated nanoparticles that could release volatile reporter molecules in response to inflammation-associated neutrophil elastase when delivered to the lungs of mice. The method achieved highly sensitive and ultrafast detection of pulmonary infection and alpha-1 antitrypsin deficiency (AATD) [115].

These studies collectively demonstrate the immense potential of synthetic biomarkers for noninvasive monitoring of organ dysfunction and injury. By designing probes that respond to the specific pathological conditions of organs, such as the gut, liver, kidney, and lung, and leveraging convenient detection media such as urine and breath, this technology offers new possibilities for early, precise diagnosis and therapeutic intervention.

### 4.5. Other Diagnostic Applications

While the applications of synthetic biomarkers in oncology, infectious diseases, and organ injuries are particularly well-established, their versatility extends to a broader spectrum of diagnostic applications, from the diagnosis of thrombosis and organ transplant rejection to therapy monitoring, such as immunotherapy and senolytic treatment.

**Figure 7:**
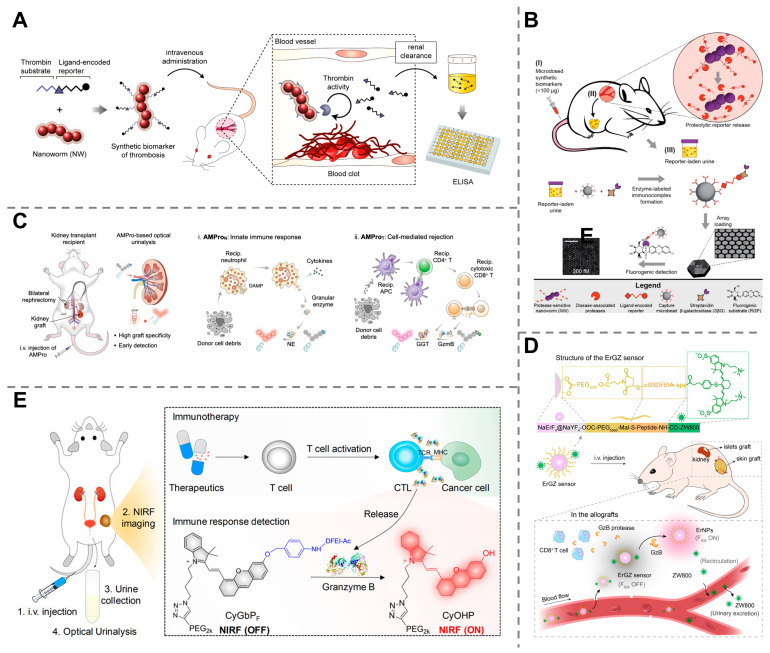
Employment of synthetic biomarkers for other diagnostic applications. (**A**) An engineered nanosensor responsive to active thrombin for noninvasive, rapid and quantitative measurement of thrombi. Reprinted with permission from Ref. [119]. Copyright 2013, American Chemical Society. (**B**) Peptide-functionalized iron oxide nanoworms for rapid diagnosis of thrombosis. Reprinted with permission from Ref. [120]. Copyright 2014, American Chemical Society. (**C**) GGT and GzmB activity-based nanoprobes allow for the discrimination of allograft rejection. Reprinted with permission from Ref. [125]. Copyright 2023, Wiley-VCH GmbH. (**D**) GzmB-activated nanoprobes for real-time evaluation of cancer immunotherapy efficacy. Reprinted with permission from Ref. [127]. Copyright 2020, American Chemical Society. (**E**) β-Gal-activated probe for monitoring the effectiveness of senolytic treatment. Reprinted with permission from Ref. [128]. Copyright 2024, Springer Nature.

Thrombosis is a serious medical condition in which blood clots form inside a blood vessel, obstructing the flow of blood to vital organs, which can be a significant health risk leading to life-threatening complications, such as stroke and heart attack [116]. Therefore, the accurate and early diagnosis of thrombosis is crucial for timely therapeutic intervention. Thrombin, an enzyme that activates the coagulation cascade and contributes to the formation of blood clots, plays a crucial role in thrombosis. Rather than the measurement of upstream or downstream byproducts of the clotting cascade, the detection of thrombin activity provides a more direct way to diagnose thrombosis [117,118]. For this purpose, activity-based synthetic nanosensors demonstrate immense potential. A protease-sensing nanosensor composed of a PEG hydrogel core and a magnetic nanoparticle shell linked with specific peptides and reporters was designed, which was cleaved by activated thrombin to liberate the reporters, making them detectable in the urine. This approach allows for noninvasive, rapid and quantitative measurement of thrombi with 100% specificity and 100% sensitivity (Figure 7A) [119]. Another similar work was reported based on the iron oxide nanoworms decorated with peptides and fluorescein-encoded reporters. Once injected into the bloodstream and localized at disease sites, the highly active thrombin cleaved the peptide substrate and released the reporters, resulting in a detectable fluorescence signal in urine in only 30 min (Figure 7B) [120].

Allograft rejection occurs during organ transplantation when the recipient’s immune system recognizes the transplanted organ as foreign and mounts an immune attack against it, leading to a slow, chronic deterioration of the organ or even an immediate organ failure, which is life-threatening to the patient and needs to be diagnosed in a timely manner. When the patient’s immune system attacks a transplanted organ, immune cells, such as neutrophils, cytotoxic T lymphocytes (CTLs), and natural killer cells (NKCs), often produce specific active proteases, for example, granzyme B (GzmB), to trigger programmed cell death [121,122,123]. An activity-based nanosensor composed of a fluorophore unit linked with a peptide was designed for early detection of acute liver allograft rejection. The nanosensor was cleaved by active GzmB and released renal-clearable fluorogenic fragments, leading to detectable fluorescence signals in urine. This technique allowed the detection of infiltration of T cells into a liver allograft as early as 24 h before clinical assays (AST, ALT, and ALP) and 72 h before histological analysis [124]. Similarly, rapid diagnosis of acute renal allograft rejection has also been achieved via the use of artificial urinary biomarkers. An artificial probe was developed to react with γ-glutamyl transpeptidase (GGT) and GzmB, specific proteases toward CTLs, leading to the activation of their near-infrared fluorescence signals that can be detected once these nanoprobes are secreted into the urine. This method has allowed for the discrimination of allograft rejection prior to histological manifestation of rejection (Figure 7C) [125]. Moreover, GzmB-responsive synthetic biomarkers have also been successfully applied in the early diagnosis of allograft rejection in skin and islet transplants [126].

Treatment monitoring is of paramount importance in modern medicine, especially for high-cost and complex treatments, such as tumor immunotherapy, which enables the timely assessment of the therapeutic efficacy to prevent unnecessary toxicity, waste of time, and waste of resources. However, traditional assessment methods, such as imaging or tissue biopsies, are often time-consuming, costly, and invasive. Synthetic biomarkers offer an unprecedented opportunity to overcome these challenges and are more suitable for precision medicine and personalized treatment regimens. For example, a near-infrared fluorescent nanoprobe, composed of a nonfluorescent dye caged by a peptide chain and linked to a PEG chain, was designed for real-time evaluation of cancer immunotherapy. When injected, these nanoprobes were cleaved by GzmB (correlated with cytotoxic T lymphocytes (CD8+) and T helper (CD4+) cells), and small fluorescent fragments were released and cleared by the kidneys, leading to detectable signals in urine. The probes demonstrated high specificity toward GzmB, allowing for real-time imaging and optical urinalysis of the immunoactivities at the tumor sites (Figure 7D) [127]. More recently, a β-Gal-activated probe was designed to detect and monitor cellular senescence, a hallmark of aging that can also be used for monitoring the effectiveness of senolytic treatment. When administered, β-Gal cleaves the bond, releasing the highly fluorescent dye, which is then excreted by the kidneys and can be measured in the urine within 30 min (Figure 7E) [128].

Throughout this section, we explored how synthetic biomarkers are revolutionizing disease diagnostics across multiple fields. By engineering smart nanoprobes that react to specific disease signals and produce a detectable output, these biomarkers enabled the detection of cancers as small as 1.6 mm, the identification of infectious diseases, and the monitoring of organ injuries. Furthermore, their versatility extends to other critical areas like the early diagnosis of thrombosis and organ transplant rejection, as well as the real-time assessment of immunotherapy and antiaging treatments. Ultimately, synthetic biomarkers demonstrated great potential to overcome the limitations of traditional diagnostic methods, providing a more sensitive, specific, and convenient way to monitor a wide range of pathological conditions.

## 5. Conclusions and Future Perspectives

Modern synthetic biomarkers have undergone accelerated advancements over the past decade, largely driven by progress in nanotechnology, which has enabled more precise targeting, enhanced signal generation, and improved biocompatibility for applications in early disease detection and diagnostics. This review thoroughly explores the current state-of-the-art of synthetic biomarkers, from their history of development and working mechanisms to their broad diagnostic applications.

Taken together, synthetic biomarkers are bioengineered nanoprobes that can respond to specific disease-related states or agents and release detectable signals, even at the very early stage when clinical symptoms have not appeared, thus addressing the limitations of endogenous biomarkers of low concentration and poor specificity. Specifically, we mainly distinguish three types of synthetic biomarkers, namely, engineered nanoparticles, activity-based probes, and genetically encoded probes, on the basis of different working mechanisms. These biomarkers have shown great promise in a variety of medical applications, particularly in the early detection of cancers, infectious diseases, organ injury, thrombosis, and allograft rejection, as well as in the assessment and monitoring of treatments, such as cancer immunotherapy and senolysis. These techniques have demonstrated great advantages over traditional biomarkers, as they allow noninvasive, highly sensitive, highly specific, rapid, and robust diagnosis.

The future of the synthetic biomarker research is bright, moving toward more quantitative, integrated and complex approaches. To ensure clinical translation, future research must first establish standardized quantitative benchmarks (including detection limits, response kinetics, and dynamic ranges) that reliably correlate synthetic biomarker signal outputs with disease progression. Creating a unified quantitative framework will not only improve the reliability and interpretability of data but also accelerate regulatory approval and cross-study validation. Second, the integration of multi-omics data (genomics, proteomics, metabolomics, and spatial biology) is crucial for providing a holistic understanding of disease processes. By identifying critical molecular pathways and enzymatic activities, multi-omics integration will significantly increase the precision of synthetic biomarker design and facilitate accurate disease diagnosis. Third, the integration with artificial intelligence (AI) and machine learning (ML) will allow for more efficient data interpretation [129,130]. On the one hand, AI algorithms can predict optimal probe architectures and simulate in vivo reaction kinetics. ML models can integrate biomarker signals with patient-level information to improve disease classification and outcome prediction. This data-driven optimization will enable personalized, adaptive, and predictive diagnostics in both clinical and home-based settings. Finally, the convergence of synthetic biomarkers with wearable diagnostic systems and point-of-care (POC) platforms holds tremendous promise for real-time, continuous, and personalized monitoring. Wearable devices, such as smart patches, flexible biosensors, and skin-integrated microfluidic collectors, can continuously sample biofluids like sweat, tears, or interstitial fluid to detect synthetic biomarker signals dynamically. These systems enable longitudinal tracking of disease biomarkers, allowing early detection of pathological changes, real-time assessment of treatment response, and timely medical intervention without hospital visits. Furthermore, such systems may not only detect disease early but also predict exacerbations, assess treatment adherence, and dynamically personalize therapy in chronic diseases like cancer, cardiovascular disorders, or autoimmune conditions. Therefore, there is a growing need to make these tools more accessible and cost-effective for wider adoption in both developed and developing regions.

However, propelling these innovative biomarkers into clinical practice is not without challenges. Safety, toxicity, biocompatibility, and ethical issues should be thoroughly evaluated and addressed before any clinical in vivo application of synthetic biomarkers. From the intricacies of their design to the complexities of their implementation, synthetic biomarkers face hurdles related to technical complexity, biocompatibility, cost and accessibility, and clinical integration.

First, the integration of synthetic biomarkers into disease diagnosis is technically challenging, especially when high sensitivity and specificity are expected. On the one hand, these biomarkers require sophisticated designs to ensure that they are specific to particular disease markers or tumor microenvironments without being influenced by other biological components or normal biological processes, to avoid inaccuracies in the diagnosis. Thus, highly adaptable design strategies for synthetic biomarkers are needed to achieve precise detection across diverse patient populations and the dynamic evolution of disease. On the other hand, establishing standardized, stable, reproducible, and scalable production is essential but poses substantial engineering challenges. This is particularly important for wearable and POC diagnostic devices, where maintaining sensor stability, consistent batch-to-batch quality, and cost-effectiveness are critical for reliable operation and clinical translation.

Another critical and significant challenge in the application of synthetic biomarkers for disease diagnosis lies in ensuring their biocompatibility and minimizing toxicity. Before being introduced into the human body, synthetic biomarkers must undergo systematic preclinical evaluation to confirm that they do not elicit adverse side effects or immune responses. Such evaluations should include good laboratory practice (GLP) toxicology and pharmacokinetic (PK) studies to assess the absorption, circulation, metabolism, and clearance pathways of synthetic biomarkers, as well as their degradation products, in order to ensure that they do not accumulate in organs like the liver or spleen. These assessments are essential because a biomarker deemed safe in one biological context may behave differently in another, depending on patient-specific factors or disease states. Addressing these concerns requires careful material design, in vitro and in vivo preclinical validation, and simulation of natural biological processes as closely as possible. Furthermore, from a regulatory standpoint, synthetic biomarkers may follow different approval pathways depending on their composition and drug classification (e.g., diagnostic agents, biologics, or diagnostic/therapeutic combination products) to ensure their biocompatibility and biodegradability. This translational process, much like drug development, is inherently costly and time-consuming.

Cost and accessibility also represent concerns that should be taken into consideration for widespread employment of synthetic biomarkers. Specifically, the process of designing, synthesizing, and rigorously testing to ensure that the developed synthetic biomarkers are both effective and safe for clinical use can be highly expensive. These costs originate from the need for advanced materials, sophisticated synthesis technology, extensive laboratory testing, and the lengthy clinical trials. The high cost of development may result in expensive diagnostic tests that could limit accessibility, particularly in low-resource regions or among populations with limited healthcare funding.

Finally, integrating synthetic biomarkers into clinical practice involves a multitude of challenges, particularly in gaining acceptance and trust from the medical community. This integration process requires not only the attestation of the superiority of synthetic biomarkers over traditional diagnostic methods and the rigorous clinical trials to validate their efficacy and safety but also the acquisition of regulatory approval, which can be both time-consuming and costly. Furthermore, healthcare providers must be trained to understand and effectively use these new tools, requiring adjustments to existing diagnostic workflows and possibly even infrastructure changes to accommodate new technologies. The entire process demands a collaborative effort among scientists, clinicians, regulatory bodies, and healthcare administrators to ensure that synthetic biomarkers can be seamlessly and beneficially incorporated into everyday medical practices.

Overall, through continued innovation, rigorous validation, and multidisciplinary collaboration, these challenges should be overcome, enabling the widespread clinical adoption of synthetic biomarkers and ushering in an era of faster, earlier, and more accurate disease detection.

## Figures and Tables

**Table 1:** Comparison of synthetic biomarkers and conventional endogenous biomarkers.

Biomarker	Potential Targets	Advantages	Disadvantages	Typical Application
Engineered nanoparticles	Disease-related physiological conditions, ROS, pH, hypoxia, metabolites	- Minimal invasiveness - Programmable targeting and signal amplification - Facile manufacturing of large-scale probes - High sensitivity and specificity - Multiplexed assays	- Potential unknown long-term toxicity - High regulatory hurdles - Early-phase trials not ready for clinical use	- Early cancer detection and diagnosis - Diagnosis of disease - Monitoring of disease activity and treatment response - Detection of metastatic spread - Monitoring of pathological processes
Activity-based probes	Enzymatic activities, redox states	- Minimal invasiveness - Fast kinetics - Mechanistic Insight for profiling enzyme activity in real-time - High Specificity to the functional activity of an enzyme	- Difficult control of delivery to desired tissue in vivo - Limited scalability in production - High regulatory hurdles - Primarily preclinical and ex vivo research in biopsy samples	
Bacteria-based genetically encoded probes	Tumor microenvironment, specific promoter activity	- Minimal invasiveness - High sensitivity due to self-amplification capability - Excellent tumor colonization	- Contaminant and biocontainment - Low public acceptance - Complex regulatory path as a live biologic - Early-phase trials not ready for clinical use	
Mammalian cell-based genetically encoded probes		- Minimal invasiveness - Human-compatible signaling - Theragnostic potential - Deep-tissue sensing - Biological amplification and high sensitivity	- High complexity and cost - High safety risks (immune rejection) - Limited scalability in production - High regulatory hurdles - Early-phase trials not ready for clinical use	
Vector-based genetically encoded probes		- Minimal invasiveness - High signal amplification capability - High flexibility and versatility	- High safety risks (toxicity, immunogenicity) - High off-target expression and background noise - Long-term persistence of vector - Significant regulatory hurdles - Early-phase trials not ready for clinical use	
Conventional endogenous biomarkers	Naturally occurring proteins, nucleic acids, cells, metabolites, exosomes	- Minimal invasiveness - Low cost and high scalability - Clarified regulatory and approval pathways - Clinically validated and current standard in diagnostics	- Low sensitivity and specificity for early disease diagnosis - High biological background noise - Limited insight into underlying active mechanism of disease progression	- Disease diagnosis - Monitoring therapy response - Risk stratification

## Data Availability

No new data were created or analyzed in this study. Data sharing is not applicable to this article.

## Abbreviations

The following abbreviations are used in this manuscript:

MRI	Magnetic resonance imaging
CT	Computed tomography
PET	Positron emission tomography
PCR	Polymerase chain reaction
NP	Nanoparticle
ABP	Activity-based probe
APN	Aminopeptidase N
CTC	Circulating tumor cell
cfDNA	Cell-free tumor DNA
V-Chip	Volumetric bar-chart chip
ROC	Receiver operating characteristic
AUC	Area under the receiver operating characteristic curve
MSC	Mesenchymal stem cell
PtNC	Platinum nanocluster
AuNC	Gold nanocluster
PEG	Poly(ethylene glycol)
AKI	Acute kidney injury
TBI	Traumatic brain injury
PPNCP	Platinum nanomarker superparticle
DTPA	Diethylenetriaminepentaacetic acid
NKC	Natural killer cell
fCAL	Fecal calprotectin
AATD	Alpha-1 antitrypsin deficiency
β-gal	Beta-galactosidase
hGluc	Humanized Gaussia luciferase
CTL	Cytotoxic T lymphocyte
GzmB	Granzyme B
GGT	γ-glutamyl transpeptidase
